# Behavioral and Neurochemical Shifts at the Hippocampus and Frontal Cortex Are Associated to Peripheral Inflammation in Balb/c Mice Infected with *Brucella abortus 2308*

**DOI:** 10.3390/microorganisms9091937

**Published:** 2021-09-11

**Authors:** José Luis Maldonado-García, Gilberto Pérez-Sánchez, Enrique Becerril Villanueva, Samantha Alvarez-Herrera, Lenin Pavón, Gabriel Gutiérrez-Ospina, Rubén López-Santiago, Jesús Octavio Maldonado-Tapia, Sonia Mayra Pérez-Tapia, Martha C. Moreno-Lafont

**Affiliations:** 1 Laboratorio de Psicoinmunología, Dirección de Investigaciones en Neurociencias del Instituto Nacional de Psiquiatría Ramón de la Fuente Muñiz, Ciudad de México 14370, Mexico; joselmgarci@comunidad.unam.mx (J.L.M.-G.); gilberto.perez.sanchez@imp.edu.mx (G.P.-S.); lusenbeve@imp.edu.mx (E.B.V.); dra.alvarezherrera@gmail.com (S.A.-H.); 2 Laboratorio de Inmunología Celular, Departamento de Inmunología, Escuela Nacional de Ciencias Biológicas, Instituto Politécnico Nacional, Ciudad de México 11340, Mexico; rlopezsantiago@hotmail.com (R.L.-S.); jmaldonadot1200@egresado.ipn.mx (J.O.M.-T.); 3 Laboratorio de Biología de Sistemas, Departamento de Biología Celular y Fisiología, Instituto de Investigaciones Biomédicas y Coordinación de Psicobiología y Neurociencias, Facultad de Psicología, Universidad Nacional Autónoma de México, Ciudad de México 04510, Mexico; gabo@biomedicas.unam.mx; 4 Unidad de Desarrollo e Investigación en Bioprocesos (UDIBI), Escuela Nacional de Ciencias Biológicas, Instituto Politécnico Nacional, Ciudad de México 11340, Mexico; smpt.2011@hotmail.com

**Keywords:** brucellosis, neurotransmitters, behavioral alterations, inflammation, brain regions

## Abstract

Brucellosis is a zoonosis affecting 50,000,000 people annually. Most patients progress to a chronic phase of the disease in which neuropsychiatric symptoms upsurge. The biological processes underlying the progression of these symptoms are yet unclear. Peripheral inflammation mounted against *Brucella* may condition neurochemical shifts and hence unchained neuropsychiatric disorders. Our work aimed at establishing whether neurological, behavioral, and neurochemical disarrays are circumstantially linked to peripheral inflammation uprise secondary to *Brucella abortus 2308* infections. We then evaluated, in control and *Brucella*-infected mice, skeletal muscle strength, movement coordination, and balance and motivation, as well as dopamine, epinephrine, norepinephrine, and serotonin availability in the cerebellum, frontal cortex, and hippocampus. Serum levels of proinflammatory cytokines and corticosterone in vehicle-injected and -infected mice were also estimated. All estimates were gathered at the infection acute and chronic phases. Our results showed that infected mice displayed motor disabilities, muscular weakness, and reduced motivation correlated with neurochemical and peripheral immunological disturbances that tended to decrease after 21 days of infection. The present observations support that disturbed peripheral inflammation and the related neurochemical disruption might lead to mood disorders in infected mice. Future experiments must be aimed at establishing causal links and to explore whether similar concepts might explain neurological and mood disorders in humans affected by brucellosis.

## 1. Introduction

Brucellosis is a disease caused by facultative intracellular, Gram-negative bacteria of the genus *Brucella* [1]. It is endemic to several regions in Africa, Asia, Latin America, and Mediterranean Europe [2,3]. Human cases of brucellosis are often caused by *B. abortus, B. melitensis, B. suis,* and *B. canis* infections. Exposure to contaminated dairy products and the manipulation of infected animals facilities the transmission and infection [1]. Unfortunately, the clinical criteria to achieve a successful diagnosis are ill-defined, and there are not enough laboratory tests to support it [4]. Since the treatment requires the patient to have a long-term commitment, relapses are rather frequent due to poor therapeutic adherence, a circumstance that challenges the effective control of brucellosis outbreaks worldwide and facilitates patients to progress to a chronic phase of the disease [5].

The clinical landscape of brucellosis is rather unspecific. Patients may report acute fever accompanied or not by a handful of signs such as headache, fatigue, weakness, overall discomfort, and tiredness. The acute stage may progress to a chronic phase that features relapses characterized by fever, weakness, and diaphoresis [6]. Chronic brucellosis may also lead to osteoarticular, skeletal muscular [7], neurological [8], and psychiatric disorders [9]. Interestingly, in patients infected by brucellosis, anxiety and depression may be manifested in the absence of demonstrable brain infection, a fact that indicates that neuropsychological symptoms may arise as a consequence of the peripheral inflammatory response mounted against it [10]. Although there is no information available to substantiate this presumption, its likeliness is supported by reports showing a correlation between IL-6 (a proinflammatory cytokine) serum concentrations and the appearance of anxiety and depression in infected patients [11]. Moreover, it has been shown that the intraperitoneal administration of proinflammatory cytokines (IL-1, TNF-α, and IL-6), alone or combined, temporally induces anhedonia [12] and that infection with bacteria of the genus *Mycobacterium* may also produce memory deficits and anhedonia [13]. The mechanisms underlying these associations, in particular those concerning *Brucella* infections, remain open issues that must be addressed. Evidence supporting that they might involve changes in neurotransmitter availability in key brain regions has been published nonetheless [12,13,14]. With this in mind, the present study was designed to evaluate circumstantial relationships between inflammatory, hormonal, neurochemical, and behavioral parameters in Balb/c mice infected or not with *Brucella abortus 2308* during the acute and chronic phase of the diseased state.

## 2. Materials and Methods

### 2.1. Bacterial Culture

The *B. abortus* strain 2308 was cultured according to a standardized method reported previously by our group [15]. Briefly, *B. abortus 2308* was cultured at 37 °C for 72 h in 10 mL trypticase soy agar supplemented with yeast extract (Merck, Cat. 146439) with constant agitation of 200× *g* at 37 °C. The bacteria were quantified by agar plate method and were adjusted by optical density using a Spectronic 20 spectrophotometer (Bausch & Lomb, Laval, Canada) with an absorbance of 0.4 ± 0.02 at 540 nm to obtain 1 × 10^6^ CFU (colony-forming units). All the procedures were performed in a Nuaire Class II type A/B3 biosafety cabinet.

### 2.2. Animals

BALB/c mice (*n* = 24) between 6 and 8 weeks of age and weighing 18–20 g were used in this study (supplied by Ferandelh, México). The mice were housed in groups of three per cage and allowed 21 days of acclimation at the animal facility of the Immunology Department at the Escuela Nacional de Ciencias Biológicas, Instituto Politécnico Nacional. Mice were kept in temperature (21 °C) and light control rooms under a regular cycle (lights on 7:00 a.m./lights off 19:00 p.m.), having free access to water and food (Lab Rodent Diet 5001, St. Louis, MO, USA) until sacrifice. Mice were randomly assigned to one of three groups. Mice in the first group (vehicle-treated or control; *n* = 12) received a single intraperitoneal injection (100 μL) of 0.1 M PBS. The other two groups (*n* = 6 each) were inoculated intraperitoneally with 100 μL PBS containing 1 × 10^6^ CFU *B. abortus 2308*. After 14 and 21 days from the infection day, the neurological and mood conditions were assessed behaviorally inside a biosafety cabinet. After being tested, mice were sacrificed by cervical dislocation, and blood and brain tissue samples were collected. Mouse handling, inoculation, and experimental protocols were revised and approved by the local Animal Rights Committee (Permit No. ZOO-009-2018) and complied fully with the NIH Guide for the Care and Use of Laboratory Animals.

### 2.3. Behavioral Assessment

To evaluate the neurological performance and mood in control and infected mice, each mouse was subjected to a battery of tests consisting of (1) a modified version of the forelimb grip strength test (FGST) [16]. Strength force was estimated in tail-pulled mice while gripped to a bar fixed to a dynamometer (Labessa, Mexico City, Mexico. Cat. DI1900) until animals released it. (2) A modified version of the motor balance and coordination test (MBCT) [17]. Mice were placed on a bar (50 cm long, 2 cm wide) placed 30 cm above the floor; the time spent traversing the bar (i.e., latency) was recorded. (3) The tail suspension test (TST) was conducted as described previously [18]. Mice were filmed for 5 min while suspended by their tails; immobilization time, defined as the moment when animals stop making paw movements, was recorded. (4) A modified version of the open field test (OF) [19]. This was conducted using a 10 × 30 cm wood-made box having its floor divided into nine quadrants. Mice were placed in the central one, and their exploratory behavior was filmed for 5 min. The number of occasions each mouse moved across quadrants was recorded. Full quadrant occupancy was recorded when the mouse posed its four paws within its limits. (5) The forced swimming test (FST) was conducted as described formerly [13]. Mice were placed in a round 1.7-L pool tempered at 35 °C and were filmed for 5 min. The immobilization time, as defined as the absence of paw movements, was recorded.

All behavioral tests were assessed in different control and infected mice (*n* = 6/group) at 14- and 21-days post-infection (PI). FGST and MBCT predominantly evaluated neurological conditions. TST, OF, and FST preferentially assessed motivation. Lastly, previous work showed that infections instrumented with *B. melitensis* did not alter blood–brain barrier permeability [20]. We then expected *B. abortus 2308* infections to cause no shifts in blood–brain barrier permeability. Indeed, unpublished results support this presumption.

### 2.4. Brain and Serum Sampling

All mice were sacrificed by cervical dislocation and later decapitated to obtain brain tissue; this procedure avoids neurochemical alterations induced by the anesthesia [21]. Once obtained, the brains, frontal cortex (FC), cerebellum, and hippocampus were dissected. All samples were weighed by using an analytical balance, frozen in liquid nitrogen, and stored at −80 °C until further processing. Peripheral blood samples, on the other hand, were withdrawn by puncturing the facial vein soon after the cervical dislocation occurred. Blood samples were centrifuged at 2000× *g* at room temperature for 10 min to separate the serum fraction. Serum aliquots (100-μL) were then collected and stored at −80 °C until use.

### 2.5. Neurotransmitter Quantification

Neurotransmitters were quantified as in a previous work [22]. Briefly, norepinephrine (NE), epinephrine (E), dopamine (D), and serotonin (5-HT) were extracted from the frontal cortex (FC), cerebellum, and hippocampus, using 400 μL extraction buffer containing 5% ascorbic acid, 200 mM sodium phosphate, 2.5 mM L-cysteine, and 2.5 mM EDTA. Then, the protein was precipitated by adding 100 μL 0.4 M perchloric acid followed by incubation at 20 °C for 20 min. Supernatants containing NE, E, D, and 5-HT were collected after centrifugation at 12,419× *g* for 10 min (4 °C). NE, E, D, and 5-HT concentrations were determined by reversed-phase HPLC (RP-HPLC) in a system integrated by a PU-2089 plus pump (Jasco, Inc. Easton, MD, USA), an AS-2057 plus autosampler (Jasco, Inc.), and an X-LC™3120FP fluorescence detector (Jasco, Inc.). Instruments were controlled using ChromNav (Jasco, Inc.). Chromatographic runs were performed using a Jupiter C18 column (300 Å, 5 μ, 4.6 × 250 mm, Phenomenex^®^) at 30 °C. The column was equilibrated with a mobile phase A containing 0.1% trifluoracetic acid in water; then, a linear gradient from min 5 to min 20 with mobile phase B containing 0.1% trifluoroacetic acid in acetonitrile was performed. Flow rate was 0.8 mL/min. Fluorescence detector was set at gain 1000, attenuation 32, response 20 s, and 280 nm and 315 nm for excitation and emission, respectively. Sample injection volume was 100 μL.

### 2.6. Cytokine Quantification

IL-6, IL-12, TNF-α, IFN-γ, and IL-10 were quantified by using a mouse inflammation kit, BD™ (catalog # 552364), used according to the manufacturer’s instruction. Samples thus processed were run in a FACSARIA III (BD Bioscience, San Jose, CA, USA) flow cytometer. Each assay was carried out in duplicate.

### 2.7. Corticosterone Quantification

Corticosterone was quantified by using the corticosterone competitive ELISA Kit (Invitrogen, cat# EIACORT) analyzed with the aid of the Multiskan Go spectrophotometer (Thermo Scientific, Waltham, MA, USA). Data were analyzed using Skanlt, version 5.0, Software for Multiskan Go; Thermo Scientific, Waltham, MA, USA, 2017.

### 2.8. Statical Analysis

All statistical analyses were performed using GraphPad Prism, version 9.0.0 for Windows, GraphPad Software, San Diego, CA, USA. Data sets were subjected to normality tests. The results were analyzed by using ANOVA or Kruskal–Wallis multiple comparisons for parametric or non-parametric data, respectively, followed by Tukey’s *post hoc* tests. Significance was established when *p* ≤ 0.05. Data sets that passed the normality tests were, for the control group, IL-6, IL-12, IFN-γ, IL-10, corticosterone; FST, MBTC, TST; and the concentration of D and E in the cerebellum; D in the frontal cortex, and NE in the hippocampus. For the 14-PI group, these were IL-12, IFN-γ, corticosterone; FST, MBTC, TST, and the concentration of D and 5-HT in the cerebellum; D, E in the frontal cortex, well as E and NE in the hippocampus. For the 21-PI group, these were IL-6, IFN-γ, corticosterone; FST, MBTC, TST; and the concentration of E, NE, and 5-HT in the cerebellum; NE, and 5-HT in the frontal cortex; and D, E and NE in the hippocampus. The rest of the data obtained did not distribute normally.

Pearson’s or Spearman’s correlation tests were used to evaluate possible correlations among cytokine serum levels, neurotransmitter profiles per anatomical brain region, corticosterone serum levels, and the behavioral parameters obtained per animal in each group. Correlation tests were conducted with data gathered either at day 14-PI (acute stage) or at day 21-PI (chronic phase). The choice of the correlation analysis depended on the result of the distribution test. Data sets collected from the three groups of mice that passed the normality tests were analyzed with Pearson’s, whereas data sets showing abnormal distributions were compared by using Spearman’s. In all cases, the *r* value ≥ 0.6, associated with a *p*-value ≥ 0.05, was considered significant and biologically meaningful.

## 3. Results

### 3.1. B. abortus 2308 Infected Mice Displayed Behavioral Changes as Compared with Control Mice at 14- and 21-Days Post-Infection

**Figure 1:**
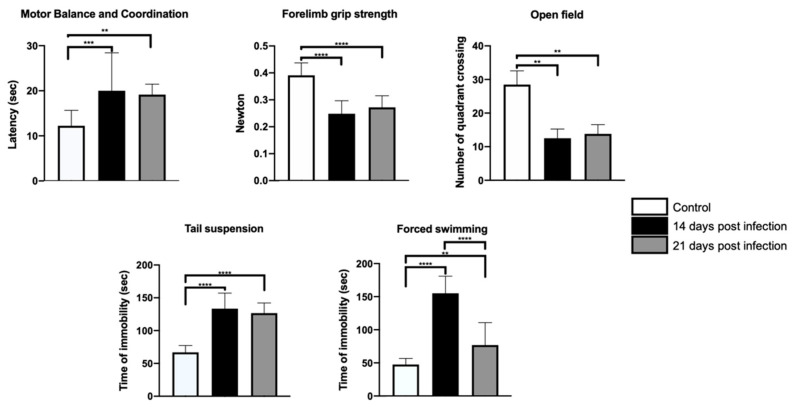
Parametrizing behavioral variables. Behavioral tests showed that the infected mice presented musculoskeletal alterations and changes in balance and motor coordination, decreased movement, and motivation. ANOVA tests were used for motor balance and coordination, tail suspension, and forced swimming tests. Kruskal–Wallis tests were used for forelimb grip strength and open field tests. Statistical significance was recognized when *p* < 0.05. Statistical significance is represented as follows: ** *p <* 0.01; *** *p* < 0.001; **** *p* < 0.0001.

*B. abortus 2308* infection disrupted mouse performance in all the behavioral tests assessed. Indeed, infected mice took more time to solve the MBCT, displayed muscle weakness in FGST, and decreased OF exploratory behavior (i.e., increased anxiety; Figure 1). Similarly, they remained immobile longer in the TST, as well as in the FST; both tests suggest a increased in hopelessness (Figure 1). The differences between control and infected mice lasted until the end of the study, with FST as exception; the behavior of hopelessness decreased between 14-PI and 21-PI days (Figure 1). Despite this finding, the magnitude of relative “hopefulness” in infected mice never quite reached the values observed in control mice (Figure 1). The differences between control and infected mice were statistically significant for FGST (*H*(2) = 83.8, *p* < 0.0001), MBCT (F = 6.6, dƒ = 2, 21; *p* < 0.0056), OF (*H*(2) = 17.4, *p* < 0.0002), TST (F = 48.5, dƒ = 2, 21; *p* < 0.0001), and FST (F = 48.4, dƒ = 2, 21; *p* < 0.0001). Table S1 shows the mean and standard deviation of the values obtained in the behavioral tests (see supplementary material).

### 3.2. B. abortus 2308 Infected Mice Display Differential Changes in Neurotransmitter Concentrations in Distinct Brain Regions as Compared with Control at 14- and 21-Days Post-Injection

**Figure 2:**
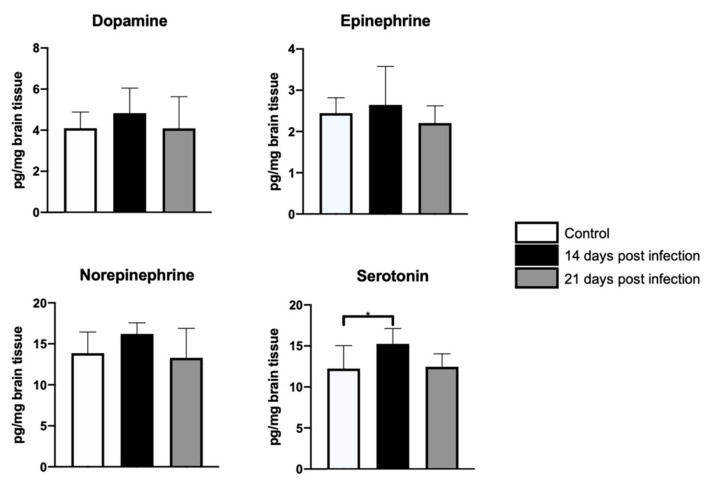
Neurotransmitter concentration in cerebellum. HPLC analyses revealed a transient, significant increase in cerebellar serotonin levels in infected mice when compared with control mice at day 14-PI. ANOVA tests were used in all cases, except for dopamine, for which Kruskal–Wallis tests were employed. Statistical significance was recognized when *p* < 0.05. Statistical significance is represented as follows: * *p* < 0.05.

**Figure 3:**
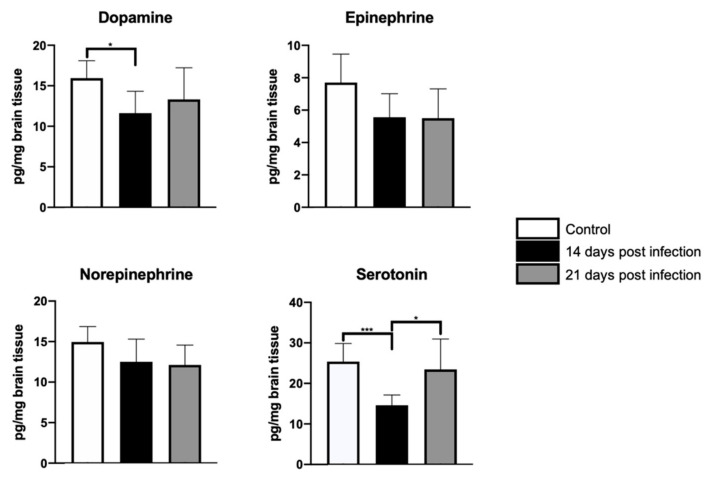
Neurotransmitter concentration in the frontal cortex. HPLC analyses documented significant decreases in frontal cortical dopamine and serotonin levels in infected mice as compared with their control mates at day 14-PI. Serotonin, nonetheless, returned to levels comparable to those observed in control mice by day 21-PI. An ANOVA test was used in all cases, except for epinephrine, in which the Kruskal–Wallis test was applied. Statistical significance was recognized when *p* < 0.05. Statistical significance is represented as follows: * *p <* 0.05; *** *p <* 0.001.

**Figure 4:**
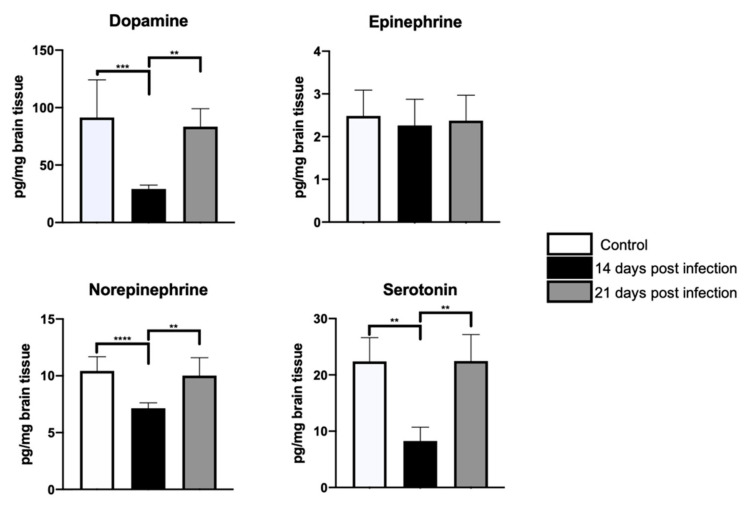
Neurotransmitter concentration in the hippocampus. HPLC analyses showed a transient drop at 14-PI, followed by a recovery at day 21-PI of hippocampal dopamine, norepinephrine, and serotonin levels in infected mice as compared with control mice. ANOVA tests were used in all cases, except for serotonin, in which the Kruskal–Wallis tests were utilized. Statistical significance was recognized when *p* < 0.05. Statistical significance is represented as follows: ** *p <* 0.01; *** *p <* 0.001; **** *p <* 0.0001.

Based upon the behavioral findings, one may expect to observe differential shifts in the availability of various neurotransmitters in cortical and subcortical areas involved in the regulation of motor and mood behavior. Accordingly, significant differences in 5-HT availability (F = 3.5, dƒ = 2, 21; *p* < 0.04), but not in dopamine (D), epinephrine (E), or norepinephrine (NE) were found between control and infected mice cerebella at day 14 post-inoculation. Figure 2 shows the result of the *post hoc* tests. A similar result was observed in the frontal cortex, where D (F = 5.1, dƒ = 2, 21; *p* = 0.01) and serotonin (5-HT) (F = 9.2, dƒ = 2, 21; *p* < 0.001) availability, but not E or NE, decreased only at 14 days-PI; the results of the *post hoc* tests are shown in Figure 3. Lastly, hippocampal D (F = 13, dƒ = 2, 21; *p* < 0.002), NE (F = 15.5, dƒ = 2, 21; *p* < 0.0001), and 5-HT (*H*(2) = 12.9, *p* < 0.001) availability, but not E concentration, significantly decreased in infected mice when compared with control mice only at day 14-PI. The values obtained and the result of the *post hoc* tests can be found in Figure 4. Tables S2–S4 shows the average and standard deviation of neurotransmitters concentration obtained in frontal cortex, hippocampus and cerebellum (see supplementary material).

### 3.3. Serum Levels of TNF-α, IL-6, IL-12, and IFN-γ at Days 14 and 21 Post-Infection with B. abortus 2308

**Figure 5:**
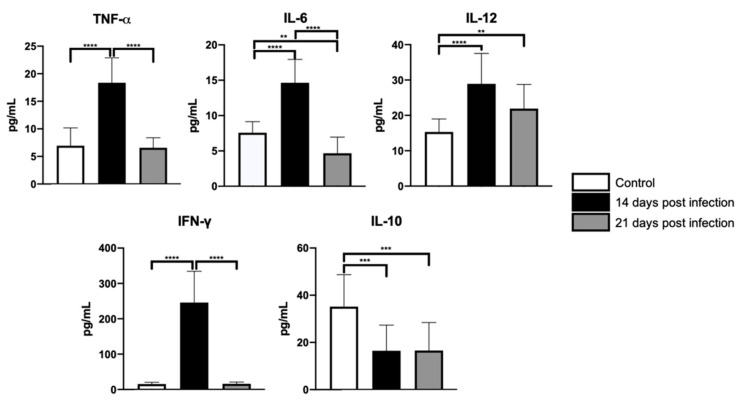
Inflammatory profile in infected Balb/c mice. A transiently exacerbated inflammatory response was documented in infected mice when compared with control mice at day 14 -PI. After this time point, the intensity of inflammatory response falls significantly by day 21-PI. Kruskal–Wallis tests were used in all cases, except for IFN-γ, in which ANOVA tests were used. Statistical significance was recognized when *p* < 0.05. Statistical significance is represented as follow: ** *p <* 0.01; *** *p* < 0.001; **** *p* < 0.0001; ns = no significance.

As previously described, infections by bacteria of the *Brucella* genus increase serum levels of proinflammatory cytokines [20]. Our observations are consistent with these results. Indeed, with the exception of IL-10 (*H*(2) = 21.78, *p <* 0.0001), we found increased serum levels of IL-6 (*H*(2) *=* 49.99, *p <* 0.0001), TNF-α (*H*(2) *=* 41.76, *p <* 0.0001), IL-12 (*H*(2) *=* 35.01, *p <* 0.0001), and IFN-γ (F *=* 161.4, dƒ = 2, 65; *p <* 0.0001) in infected mice when compared with the control ones at day 14-PI. In contrast, at day 21-PI, the only cytokine whose concentration was found elevated in infected mice was IL-12. Interestingly, also at day 21-PI, IL-6 and IL-10 serum levels decreased significantly in infected mice as compared with their control peers. The results of the comparisons between groups are shown in Figure 5. Table S5 shows the mean and standard deviation of cytokine concentration quantified in peripheral blood (see supplementary material).

### 3.4. B. abortus 2308 Infected Mice Did Not Display Differential Changes in Corticosterone Serum Concentrations as Compared Control Mice

Corticosterone levels did not show changes in the infected versus control ones at 14- and 21-days PI (F = 2.5, dƒ = 2, 45; *p >* 0.05). This recalls the phenomenon observed in patients with brucellosis, who showed no changes in blood cortisol levels during infection, but who did have a decrease in serum dehydroepiandrosterone levels. It has been suggested that the infection modifies the synthesis of dehydroepiandrosterone as a mechanism of evasion of the immune response [23].

### 3.5. Correlations between Cytokines, Corticosterone, Neurotransmitters, and Behavioral Tests

To evaluate possible interactions between cytokine and corticosterone peripheral levels, concentrations of neurotransmitters in different brain regions, and the values obtained for each behavioral parameter evaluated, we constructed a correlation matrix that was analyzed by using Spearman’s tests for data having Gaussian’s distribution or Pearson’s correlation tests for data displaying non-normal distribution. The results are shown in Table 1, Table 2, Table 3 and Table 4. Overall, our results show that neurotransmitter concentrations across the anatomical regions evaluated correlated with inflammatory, hormone, and behavioral parameters, as evaluated from one time point to the other in infected mice. The number of correlations was greater in 21-PI mice, thus, suggesting that the neurochemical disturbances and, hence, mood and motor disorders are greater as the inflammatory response develops further from 14 to 21 days PI.

## 4. Discussion

It is estimated that approximately one-third of the patients affected by chronic infectious diseases develop psychiatric conditions, even in the absence of demonstrable neural infections [24,25,26]. Patients chronically infected by *Brucella* are not an exception [11]. Even though the mechanisms underlying the neuropsychological manifestations of brucellosis are unclear, the rise in circulatory levels of cytokines and microbial-derived antigens may alter the body’s neuro-immune balance in the body [27,28] and, thus, the behavior [29,30].

### 4.1. Inflammatory Changes

As expected, the inoculation of *B. abortus 2308* increased serum levels of proinflammatory cytokines IL-6, IL-12, TNF-α, and IFN-γ at day 14-PI [20]. The levels of the majority of these cytokines, with the exception of IL-12, decreased by day 21-PI. In the case of IFN-γ, the drop in its serum levels by day 21-PI is reminiscent of the kinetics observed for this cytokine in chronic *Brucella*-infected patients [31]. The decrease in IFN-γ serum levels observed in mice and humans infected by *Brucella* may in part be explained by the reduction in the macrophage number in the splenic marginal zone [32].

In our experimental unit, based upon a two-point inference model and the concentration of the circulating levels of IFN-γ, we identified a two-phase immune response mounted against *Brucella*. During the primary phase that lasts a couple of weeks, an intense inflammatory response accompanied by increments in IFN-γ serum levels was observed. In contrast, the second phase, developed between day 14-PI and day 21-PI, was characterized by a drop in IFN-γ serum and a decreased intensity of the immune response. This dynamic is consistent with that reported previously by Demirdag et al. [33] and Ghaznavi-Rad et al. [31] and suggests that *Brucella* evades the immune response when the infection becomes chronic.

An interesting finding made in our work is the sustained increase in IL-12 accompanied by the decay of IL-10 serum concentrations in *Brucella*-infected mice. Although IL-12 and IL-10 are considered essentially antagonistic, under the context of *Brucella* infections, both cytokines may work in concert to limit the progression of the infection. Indeed, on the one hand, IL-12 is a proinflammatory cytokine produced by macrophages and other antigen-presenting cells, among other functions, of inducing IFN-γ production and promoting Th1 lymphocyte activation [34]. These IL-12 properties help to organize immune responses aimed at clearing off intracellular microorganisms infections [35], among them *Brucella* [36]. IL-10, on the other hand, is a potent anti-inflammatory agent that prevents tissue damage by the immune response [37]. In the case of *Brucella* infections, this property facilitates the inflammatory response against it [38], likely ameliorating the severity of tissue damage.

### 4.2. Corticosterone

In our experimental model, we did not detect changes in plasma corticosterone levels. A similar observation has been reported in patients infected with *Brucella*. It has been revealed, nonetheless, that *Brucella* may evade the immune response after inducing increments of dehydroepiandrosterone [23]. Future studies will evaluate this possibility in infected mice.

### 4.3. Neurochemical Alterations

Since the permeability of the blood–brain barrier remains intact in *Brucella* inoculated Balb/c mice [20,39], we assumed that their behavioral and neurochemical disturbances occur as a consequence of the peripheral inflammatory response. In support of this notion, it has been reported that brucellosis-infected patients develop, during the acute phase of the illness, behavioral and mood clinical manifestations such as hallucinations, *delirium*, or psychotic symptoms [10,11]. Depression, on the other hand, is not infrequent in patients suffering chronic brucellosis [10].

To date, the mechanism by which *Brucella* infection leads to altered neural function is yet unknown. Since bacterial invasion to neural tissue has not been demonstrated, the release of bacterial components upon peripheral nerve endings and/or centrally on brain-specific regions constitutes one possibility [40,41]. Even though our results do not provide definitive answers to this conundrum, they do support that brucellosis infections differentially affect the neurochemistry across the brain. Indeed, we observed, for example, decreased D and 5-HT levels in the frontal cortex and reduced D, NE, and 5-HT concentrations in the hippocampus at day 14-PI. These results are in line with previous reports documenting differential neurochemical disturbances secondary to infections produced by other intracellular microorganisms such as *Mycobacterium lepraemurium* [14], *Mycobacterium tuberculosis* [13], and *Bacille Calmette Guérin* [42]. Hence, brain differential regional neurochemical responses seem to be a feature by virtue of which the brain contends extraneuronal infections produced by intracellular bacteria. Whether this mosaic response has an adaptive value and/or whether it represents an iterated code developed by the brains of hosts specifically infected by intracellular microorganisms await to be investigated.

### 4.4. Behavioral Alterations

Our results showed that infected mice develop motor disabilities, muscular weakness, and reduced motivation, as monitored by various neurological and mood tests at 14- and 21 -days PI. These behavioral disturbances persisted despite the relative “normalization” of the concentration of inflammatory cytokines and neurotransmitter availability with time. This observation suggests that brain damage may be long-lasting. In the future, we must address whether this assertion is fair and whether the long-term plastic response is indeed insufficient to fully restore neural function in infected mice. That this might be the case, however, is supported by the observation showing functional *sequela* in patients having chronic brucellosis [10,11]. Lastly, although there are no reports of behavioral changes in murine models following infection with *B. abortus 2308*, there is evidence that infections by other microorganisms indeed cause behavioral disturbances [13,14].

A finding that deserves special consideration is that showing that infected mice exhibited almost identical increased immobility times at 14-PI and 21-PI days while the levels of IFN-γ were bell-shaped. Since IFN-γ levels are frequently thought to be positively correlated with depression, one might have expected IFN-γ levels to be similar in these animal groups. Recent studies have shown, nonetheless, that IFN-γ [43] levels are lower in patients diagnosed with major depression than in treated, depressed patients or healthy volunteers [44], thus suggesting that the relationship between depressive symptoms and IFN-γ levels is not as straightforward as previously thought. In fact, it might be modulated by interactions with other proinflammatory cytokines (e.g., IL-1, TNF-α, and IL-6; [12,45]), hormones, and neurotransmitters [46,47,48,49]. Therefore, future studies must be designed to disentangle the interactions ongoing within this network (for an initial guide, see Table 5 in the discussion) to begin understanding how depression is installed in individuals carrying chronic brucellosis.

### 4.5. Correlations

In our analysis, we highlight some correlations. At day 14-PI the correlations detected were between the peripheral IL-12 levels and NE concentrations in the frontal cortex (*r* = 0.61; *p <* 0.05); as well as a negative correlation between IL-10 and D levels in the frontal cortex (*r* = −0.77; *p <* 0.01). This suggests a strong influence of the inflammatory response on changes in neurotransmitter concentrations. Additionally, we found a strong correlation (*r_s_* = 0.95 *p <* 0.05) between TNF-α levels and those of corticosterone. This is likely the result of an evasion mechanism of the host’s immune response against the microorganism [23]. A possible explanation of this phenomenon could be the result of the deregulation of the vagal anti-inflammatory pathway since the TNF-α and cortisol values detected in our model suggest the presence of a reduced cholinergic tone [46].

At day 21-PI we highlight a positive correlation between circulatory levels of IL-6 and D in the cerebellum (*r* = 0.75; *p <* 0.01), and serotonin in the hippocampus (*r* = 0.78; *p <* 0.01). Such changes could result from a decrease in proinflammatory cytokines that induce a reduction in IDO activity, which favors a compensation in neurotransmitter levels [47,48] compared against observations at day 14. There is a relevant correlation between the forelimb grip strength test and D in the frontal cortex (*r_s_* = −0.76; *p <* 0.01) and serum IL-10 levels (*r_s_* = −0.65; *p <* 0.05) that could be explained by the participation of MPFC and the cerebellum in executive functions through a dopaminergic axis [49,50]. The abundant neurochemical changes observed at day 21 in the frontal cortex, hippocampus, and cerebellum of infected animals are similar to those observed in human and animal models regarding their participation in cognitive-affective functions [51,52].

In sum, the overall results (Table 5) support that brucellosis might produce neurological and motivational disturbances by acting, not directly but indirectly, via the body’s peripheral inflammatory response. That this might be possible is supported by data showing that intraperitoneal injection of single or combination proinflammatory cytokines (IL-1β, IL-6 and TNF-α) influence neuroendocrine activity, which promotes sickness behavior, anxiety, and anhedonia accompanied by neurotransmitter alterations in different brain areas such as the amygdala, hippocampus, locus coeruleus, and medial prefrontal cortex; sickness behavior persisted for 24 h after cytokine administration [12]. In the same way, our group reported alterations in hippocampal NE concentration 24 h after LPS injection in Wistar rats [53]. In addition, we have claimed that neurotransmitter shifts in the hippocampus and frontal cortex might be secondary to the effect of inflammatory cytokines on the activity of indoleamine 2,3-dioxygenase (IDO) [48,54] and tetrahydrobiopterin (BH4) [55]; activation of IDO decreases serotonin synthesis [48]. On the other hand, inflammation inhibits BH4 and decreases dopamine and monoamine synthesis [55]. In the cerebellum, we observed only increments in 5-HT levels at day 14-PI. This increment might be explained by the local increment of melatonin, a neuroprotective response against noxious stimuli instrumented by the cerebellum [56].

### 4.6. Limitations

The correlative design of the present work does not permit readers to draw conclusive cause-effect relationships. The relatively small sample size makes this work exploratory and preliminary in nature. The lack of biochemical information on the striatum and amygdala limits, to some extent, the conclusions reached with regard to the behavioral outcomes observed in infected mice.

## 5. Conclusions

Having these limitations in mind, we think the results presented here are sufficient to conjecture that the peripheral inflammatory response mounted against *B. abortus 2308* explains both the neurological and mood disorders observed in the infected mice. If a similar association is uncovered and demonstrated to be causal in humans, future clinical protocols aimed at managing chronic brucellosis must contemplate psychoneuroimmune screening.

## Figures and Tables

**Table 1:** Correlative analyses among parametrized variables in control mice.

Cerebellum			
Neurotransmitter	Correlation	Correlation Value	*p*-Value
D	FGST	*r_s_* = 0.67	*p <* 0.05
	MBCT	*r* = −0.76	*p <* 0.01
E	Cx	*r =* 0.79	*p <* 0.01
**Frontal Cortex**			
D	MBCT	*r=* −0.62	*p <* 0.05
**Hippocampus**			
**NE**	OF	*r_s_ =* 0.67	*p <* 0.01

Correlation values are presented with *r* for Pearson correlation and *r_s_* for Spearman correlation. MBCT, motor balance and coordination test; FGST, forelimb grip strength test; OF, open field test; Cx, corticosterone; FC, frontal cortex; H, hippocampus.

**Table 2:** Correlative analyses among parametrized variables in infected mice at day 14-PI.

Frontal Cortex			
Neurotransmitter	Correlation	Correlation Value	*p*-Value
D	IL-10	*r_s_ =* −0.77	*p <* 0.01
E	NE FC	*r_s_ =* 0.71	*p <* 0.01
**NE**	IL-12	*r =* 0.61	*p <* 0.05
**Hippocampus**			
D	MBCT	*r =* 0.74	*p <* 0.01

Correlation values are presented with *r* for Pearson correlation and *r_s_* for Spearman correlation. FC, frontal cortex; MBCT, motor balance and coordination test.

**Table 3:** Correlative analyses among parametrized variables in infected mice at day 21-PI.

Cerebellum			
Neurotransmitter	Correlation	Correlation Value	*p*-Value
D	IL-6	*r =* 0.75	*p <* 0.01
**Frontal Cortex**			
D	FGST	*r_s_=* −0.76	*p <* 0.01
E	Cx	*r_s_=* 0.62	*p <* 0.05
**NE**	FST	*r =* −0.6	*p <* 0.05
	IL-10	*r_s_=* −0.7	*p <* 0.01
**5**-**HT**	TST	*r =* 0.77	*p <* 0.01
	FST	*r =* −0.61	*p <* 0.05
**Hippocampus**			
D	IL-6	*r =* 0.77	*p <* 0.01
E	TST	*r =* 0.58	*p <* 0.05
**NE**	MBCT	*r =* 0.61	*p <* 0.05
	TST	*r =* 0.60	*p <* 0.05

Correlation values are represented with *r* for Pearson correlation and *r_s_* for Spearman correlation. MBCT, motor balance and coordination test; FGST, forelimb grip strength test; FGST, forelimb grip strength test; FST, forced swimming test; TST, tail test suspension; FC, frontal cortex; H, hippocampus.

**Table 4:** Correlative analyses between cytokines and other parametrized variables across infected mice.

Cytokine	Correlation	Correlation Value	*p*-Value
**IL-6** (**21**-PI)	Cx (21-PI)	*r =* 0.68	*p <* 0.05
**IL-10** (21-PI)	FGST (21-PI)	*r_s_=* 0.65	*p <* 0.05
**TNF-α** (**14**-PI)	Cx (14-PI)	*r_s_=* 0.95	*p <* 0.001

Correlation values are presented with *r* for Pearson correlation and *r_s_* for Spearman correlation. FGST, forelimb grip strength test; OF, open field test; Cx, corticosterone.

**Table 5:** Overall cytokine, neurotransmitter and behavioral changes observed in mice infected with *Brucella abortus* 2308.

Molecule	14-PI		21-PI	
Cytokines	TNF-α	↑	TNF-α	↑ ↓
	IL-6	↑	IL-6	↑ ↓
	IL-12	↑	IL-12	↑
	IL-10	↓	IL-10	↓
	IFN-γ	↑	IFN-γ	↓ ↓
Neurotrasmitters	Hippocampus			
	5-HT	↓	5-HT	↓ ↑
	D	↓	D	↓ ↑
	NE	↓	NE	↓ ↑
	Frontal cortex			
	5-HT	↓	5-HT	↓ ↑
	D	↓		
	Cerebellum			
	5-HT	↑		
Hormones	Cortisol =		Cortisol =	
Behavior	Resistance (FGST)	↓	Resistance (FGST)	↓
	Equilibrium (MBCT)	↓	Equilibrium (MBCT)	↓
	Anxiety (OF)	↑	Anxiety (OF)	↑
	Hopelessness (TST)	↓	Hopelessness (TST)	↓
	Hopelessness (FST)	↓	Hopelessness (FST)	↓ ↑

Blue arrows indicate comparison with control group; red arrows indicate comparison with 14-PI mice group; PI, post-infection; =, indicate no changes in comparison with control group; FST, forced swimming test; MBCT, motor balance and coordination test; FGST, forelimb grip strength test; OF, open field test; TST, tail suspension test.

## Data Availability

Data are contained within the article and in supplementary material.

## Supplementary Materials

The following are available online at https://www.mdpi.com/article/10.3390/microorganisms9091937/s1, Table S1: Results of the behavioral tests performed in the different groups. Table S2: Concentration of neurotransmitters in cerebellum Table S3: Concentration of neurotransmitters in frontal cortex. Table S4: Concentration of neurotransmitters in the hippocampus. Table S5: Inflammatory profile in control and infected mice. In all cases standard deviation are included.

## References

[1] Hayoun M., Muco E., Shorman M. (2020). StatPearls.

[2] Adetunji S.A., Ramirez G., Foster M.J., Arenas-Gamboa A.M. (2019). A systematic review and meta-analysis of the prevalence of osteoarticular brucellosis. PLoS Negl. Trop. Dis..

[3] Roushan M.R.H., Ebrahimpour S. (2015). Human brucellosis: An overview. Casp. J. Intern. Med..

[4] Castaño M.J., Solera J. (2009). Chronic brucellosis and persistence of *Brucella melitensis* DNA. J. Clin. Microbiol..

[5] Ariza J., Bosilkovski M., Cascio A., Colmenero J.D., Corbel M.J., Falagas M.E., Memish Z.A., Roushan M.R.H., Rubinstein E., Sipsas N.V. (2007). Perspectives for the Treatment of Brucellosis in the 21st Century: The Ioannina Recommendations. PLoS Med..

[6] Franco M.P., Mulder M., Gilman R.H., Smits H.L. (2007). Human brucellosis. Lancet Infect. Dis..

[7] Buzgan T., Karahocagil M.K., Irmak H., Baran A.I., Karsen H., Evirgen O., Akdeniz H. (2010). Clinical manifestations and complications in 1028 cases of brucellosis: A retrospective evaluation and review of the literature. Int. J. Infect. Dis..

[8] Adeva-Bartolomé M.T., Montes-Martínez I., Castellanos-Pinedo F., Zurdo-Hernández J.M., De Castro-García F.J. (2005). Neurobrucellosis: Four case reports. Rev. Neurol..

[9] Shoaei S.D., Bidi N. (2012). Serologic evaluation of brucellosis in patients with psychiatric disorders. Casp. J. Intern. Med..

[10] Mufaddel A., Omer A.A., Salem M.O. (2014). Psychiatric Aspects of Infectious Diseases. Open J. Psychiatry.

[11] Shehata G.A., Abdel-Baky L., Rashed H., Elamin H. (2010). Neuropsychiatric evaluation of patients with brucellosis. J. Neurovirol..

[12] Brebner K., Hayley S., Zacharko R., Merali Z., Anisman H. (2000). Synergistic effects of interleukin-1β, interleukin-6, and tumor necrosis factor-α: Central monoamine, corticosterone, and behavioral variations. Neuropsychopharmacology.

[13] Lara-Espinosa J.V., Santana-Martínez R.A., Maldonado P.D., Zetter M., Becerril-Villanueva E., Pérez-Sánchez G., Pavón L., Mata-Espinosa D., Barrios-Payán J., López-Torres M.O. (2020). Experimental pulmonary tuberculosis in the absence of detectable brain infection induces neuroinflammation and behavioural abnormalities in male balb/c mice. Int. J. Mol. Sci..

[14] Becerril-Villanueva E., Ponce-Regalado M.D., Pérez-Sánchez G., Salazar-Juárez A., Arreola R., Álvarez-Sánchez M.E., Juárez-Ortega M., Falfán-Valencia R., Hernández-Pando R., Morales-Montor J. (2018). Chronic infection with Mycobacterium lepraemurium induces alterations in the hippocampus associated with memory loss. Sci. Rep..

[15] Paredes-Cervantes V., Flores-Mejía R., Moreno-Lafont M.C., Lanz-Mendoza H., Tello-López Á.T., Castillo-Vera J., Pando-Robles V., Hurtado-Sil G., González-González E., Rodríguez-Cortés O. (2011). Comparative proteome analysis of *Brucella abortus 2308* and its virB type IV secretion system mutant reveals new T4SS-related candidate proteins. J. Proteomics.

[16] Gibbs E.M., Crosbie-Watson R.H. (2017). A simple and low-cost assay for measuring ambulation in mouse models of muscular dystrophy. J. Vis. Exp..

[17] Luong T.N., Carlisle H.J., Southwell A., Patterson P.H. (2011). Assessment of motor balance and coordination in mice using the balance beam. J. Vis. Exp..

[18] Can A., Dao D.T., Terrillion C.E., Piantadosi S.C., Bhat S., Gould T.D. (2011). The tail suspension test. J. Vis. Exp..

[19] Tatem K.S., Quinn J.L., Phadke A., Yu Q., Gordish-Dressman H., Nagaraju K. (2014). Behavioral and locomotor measurements using an open field activity monitoring system for skeletal muscle diseases. J. Vis. Exp..

[20] Osman A.Y., Kadir A.A., Jesse F.F., Saharee A.A. (2019). Modelling the immunopathophysiology of *Brucella melitensis* and its lipopolysaccharide in mice infected via oral route of exposure. Microb. Pathog..

[21] Son Y. (2010). Molecular mechanisms of general anesthesia. Korean J. Anesthesiol..

[22] Mendieta I., Nuñez-Anita R.E., Pérez-Sánchez G., Pavón L., Rodríguez-Cruz A., García-Alcocer G., Berumen L.C. (2018). Effect of A549 neuroendocrine differentiation on cytotoxic immune response. Endocr. Connect..

[23] Gentilini M.V., Velásquez L.N., Barrionuevo P., Benitez P.C.A., Giambartolomei G.H., Delpino M.V. (2015). Adrenal steroids modulate the immune response during *brucella abortus* infection by a mechanism that depends on the regulation of cytokine production. Infect. Immun..

[24] Kronfol Z., Remick D.G. (2000). Cytokines and the brain: Implications for clinical psychiatry. Am. J. Psychiatry.

[25] Bonaccorso S., Marino V., Biondi M., Grimaldi F., Ippoliti F., Maes M. (2002). Depression induced by treatment with interferon-alpha in patients affected by hepatitis C virus. J. Affect. Disord..

[26] Taquet M., Luciano S., Geddes J.R., Harrison P.J. (2021). Bidirectional associations between COVID-19 and psychiatric disorder: Retrospective cohort studies of 62 354 COVID-19 cases in the USA. Lancet Psychiatry.

[27] Miller A.H., Haroon E., Raison C.L., Felger J.C. (2013). Cytokine targets in the brain: Impact on neurotransmitters and neurocircuits. Depress. Anxiety.

[28] Dantzer R. (2009). Cytokine, Sickness Behavior, and Depression. Immunol. Allergy Clin. N. Am..

[29] Pavlov V.A., Tracey K.J. (2017). Neural regulation of immunity: Molecular mechanisms and clinical translation. Nat. Neurosci..

[30] Salvador A.F., de Lima K.A., Kipnis J. (2021). Neuromodulation by the immune system: A focus on cytokines. Nat. Rev. Immunol..

[31] Ghaznavi-Rad E., Khosravi K., Zarinfar N., Mosayebi G. (2017). Reduced IFN-γ production in chronic brucellosis patients. Iran. J. Immunol..

[32] Machelart A., Khadrawi A., Demars A., Willemart K., de Trez C., Letesson J.J., Muraillec E. (2017). Chronic *Brucella* infection induces selective and persistent interferon gammadependent alterations of marginal zone macrophages in the spleen. Infect. Immun..

[33] Demirdag K., Ozden M., Kalkan A., Godekmerdan A., Kilic S.S. (2003). Serum cytokine levels in patients with acute brucellosis and their relation to the traditional inflammatory markers. FEMS Immunol. Med. Microbiol..

[34] Zundler S., Neurath M.F. (2015). Interleukin-12: Functional activities and implications for disease. Cytokine Growth Factor Rev..

[35] Romani L., Puccetti P., Bistoni F. (1997). Interleukin-12 in infectious diseases. Clin. Microbiol. Rev..

[36] Zhan Y., Cheers C. (1995). Endogenous interleukin-12 is involved in resistance to *Brucella abortus* infection. Infect. Immun..

[37] Iyer S.S., Cheng G. (2012). Role of interleukin 10 transcriptional regulation in inflammation and autoimmune disease. Crit. Rev. Immunol..

[38] Corsetti P.P., de Almeida L.A., Carvalho N.B., Azevedo V., Silva T.M.A., Teixeira H.C., Faria A.C., Oliveira S.C. (2013). Lack of Endogenous IL-10 Enhances Production of Proinflammatory Cytokines and Leads to *Brucella abortus* Clearance in Mice. PLoS ONE.

[39] Hurtado-Alvarado G., Becerril-Villanueva E., Contis-Montes de Oca A., Domínguez-Salazar E., Salinas-Jazmín N., Pérez-Tapia S.M., Pavon L., Velázquez-Moctezuma J., Gómez-González B. (2018). The yin/yang of inflammatory status: Blood-brain barrier regulation during sleep. Brain. Behav. Immun..

[40] Dmitrieva N., Rodríguez-Malaver A.J., Pérez J., Hernández L. (2004). Differential release of neurotransmitters from superficial and deep layers of the dorsal horn in response to acute noxious stimulation and inflammation of the rat paw. Eur. J. Pain.

[41] Lasselin J., Schedlowski M., Karshikoff B., Engler H., Lekander M., Konsman J.P. (2020). Comparison of bacterial lipopolysaccharide-induced sickness behavior in rodents and humans: Relevance for symptoms of anxiety and depression. Neurosci. Biobehav. Rev..

[42] Rodriguez-Zas S.L., Nixon S.E., Lawson M.A., Mccusker R.H., Southey B.R., O’Connor J.C., Dantzer R., Kelley K.W. (2015). Advancing the understanding of behaviors associated with Bacille Calmette Guérin infection using multivariate analysis. Brain Behav. Immun..

[43] Filiano A.J., Xu Y., Tustison N.J., Marsh R.L., Baker W., Smirnov I., Overall C.C., Gadani S.P., Turner S.D., Weng Z. (2016). Unexpected role of interferon-γ in regulating neuronal connectivity and social behaviour. Nature.

[44] Hernández M.E., Mendieta D., Martínez-Fong D., Loría F., Moreno J., Estrada I., Bojalil R., Pavón L. (2008). Variations in circulating cytokine levels during 52 week course of treatment with SSRI for major depressive disorder. Eur. Neuropsychopharmacol..

[45] Dunn A.J., Swiergiel A.H., Beaurepaire R. (2005). De Cytokines as mediators of depression: What can we learn from animal studies?. Neurosci. Biobehav. Rev..

[46] Pellissier S., Dantzer C., Mondillon L., Trocme C., Gauchez A.-S., Ronique Ducros V., Mathieu N., Toussaint B., Fournier A., dé ric Canini F. (2014). Relationship between Vagal Tone, Cortisol, TNF-Alpha, Epinephrine and Negative Affects in Crohn’s Disease and Irritable Bowel Syndrome. PLoS ONE.

[47] Tu H., Rady P.L., Juelich T., Smith E.M., Tyring S.K., Hughes T.K. (2005). Cytokine regulation of tryptophan metabolism in the hypothalamic-pituitary- adrenal (HPA) axis: Implications for protective and toxic consequences in neuroendocrine regulation. Cell. Mol. Neurobiol..

[48] Kim H., Chen L., Lim G., Sung B., Wang S., McCabe M.F., Rusanescu G., Yang L., Tian Y., Mao J. (2012). Brain indoleamine 2,3-dioxygenase contributes to the comorbidity of pain and depression. J. Clin. Investig..

[49] Giompres P., Delis F. (2005). Dopamine transporters in the cerebellum of mutant mice. Cerebellum.

[50] Rogers T.D., Dickson P.E., McKimm E., Heck D.H., Goldowitz D., Blaha C.D., Mittleman G. (2013). Reorganization of circuits underlying cerebellar modulation of prefrontal cortical dopamine in mouse models of autism spectrum disorder. Cerebellum.

[51] Heller A.S. (2016). Cortical-subcortical interactions in depression: From animal models to human psychopathology. Front. Syst. Neurosci..

[52] Depping M.S., Schmitgen M.M., Kubera K.M., Wolf R.C. (2018). Cerebellar Contributions to Major Depression. Front. Psychiatry.

[53] Nachón-García F., Hurtado-Alvarado G., Acosta-Hernández M.E., Peña-Escudero C., Priego-Fernández S., Alvarez-Herrera S., Becerril-Villanueva E., Pérez-Sánchez G., Pavón L., García-García F. (2018). Characterization of sleep-pattern and neuro-immune-endocrine markers at 24 hour post-injection of a single low dose of lipopolysaccharide in male Wistar rats. J. Neuroimmunol..

[54] Qin Y., Wang N., Zhang X., Han X., Zhai X., Lu Y. (2018). IDO and TDO as a potential therapeutic target in different types of depression. Metab. Brain Dis..

[55] Vancassel S., Capuron L., Castanon N. (2018). Brain Kynurenine and BH4 Pathways: Relevance to the Pathophysiology and Treatment of Inflammation-Driven Depressive Symptoms. Front. Neurosci..

[56] Pinato L., da Silveira Cruz-Machado S., Franco D.G., Campos L.M.G., Cecon E., Fernandes P.A.C.M., Bittencourt J.C., Markus R.P. (2015). Selective protection of the cerebellum against intracerebroventricular LPS is mediated by local melatonin synthesis. Brain Struct. Funct..

