# Supplementary material for: Behavioral and Neurochemical Shifts at the Hippocampus and Frontal Cortex Are Associated to Peripheral Inflammation in Balb/c Mice Infected with Brucella abortus 2308
Source: Microorganisms. 2021 Sep 11;9(9):1937. doi: 10.3390/microorganisms9091937 (PMC8470318; doi:10.3390/microorganisms9091937)
Supplement: Supplementary file 1 [file microorganisms-09-01937-s001.zip › microorganisms-1340809-supplementary.pdf]

Supplementary material

Table with values from behavioral test and cytokines results which complements figures 1-5.

Table S1. Behavorial test results in Balb/c infected with *B. abortus* 2308

**Motor Balance and Coordination Test**						
	**Groups**			***post hoc* test**		
	Control n=12	14-PI n=6	21-PI n=6	14-PI vs. Control	21-PI vs. Control	14-PI vs. Control
Time (s)	12.2 ± 3.4	20 ± 8.0	19.1 ± 2.2	***	**	ns
**Forelimb grip strength test**						
	**Groups**			***post hoc* test**		
	Control n=12	14-PI n=6	21-PI n=6	14-PI vs. Control	21-PI vs. Control	14-PI vs. Control
Resistence (N)	0.39 ± 0.04	0.24 ± 0.04	0.27 ± 0.04	****	****	ns
**Open field test**						
	**Groups**			***post hoc* test**		
	Control n=12	14-PI n=6	21-PI n=6	14-PI vs. Control	21-PI vs. Control	14-PI vs. Control
Changes in square	28.5 ±4.0	12.5 ± 2.6	13.83 ± 2.5	**	**	ns
**Tail suspension test**						
	**Groups**			***post hoc* test**		
	Control n=12	14-PI n=6	21-PI n=6	14-PI vs. Control	21-PI vs. Control	14-PI vs. Control
Time (s)	66.92 ± 10	133.3 ± 22	126.5 ± 14	****	****	ns
**Forced swimming test**						
	**Groups**			***post hoc* test**		
	Control n=12	14-PI n=6	21-PI n=6	14-PI vs. Control	21-PI vs. Control	14-PI vs. Control
Time (s)	47.5 ± 9.1	155 ± 24	76.8 ± 32	****	**	****

In all cases the mean ± standard deviation is described. 14-PI= 14 days post infection, 21-PI= 21 days post infection. ANOVA tests were used for motor balance and coordination, tail suspension, and forced swimming tests. Kruskal–Wallis tests were used for forelimb grip strength and open field tests. Statistical significance was recognized when $p < 0.05$. Statistical significance is represented as follows: * $p < 0.05$; ** $p < 0.01$; *** $p < 0.001$; **** $p < 0.0001$.

Table S2. Determination of neurotransmitters in the cerebellum

	Groups			*Post hoc* test		
Neurotransmitter (pg/mL)	Control n=12	14-PI n=6	21-PI n=6	14-PI vs. Control	21-PI vs. Control	14-PI vs. Control
D	4 ± 0.7	4.8 ± 1.2	4 ± 1.5	ns	ns	ns
E	2.4 ± 0.3	2.6 ± 0.9	2.2 ± 0.4	ns	ns	ns
NE	13.8 ± 2.5	16.2 ± 1.3	13.2 ± 3.5	ns	ns	ns
5-HT	12.2 ± 2.7	15.2 ± 1.8	12.4 ± 1.5	*	ns	ns

In all cases the mean ± standard deviation is described. 14-PI= 14 days post infection, 21-PI= 21 days post infection. ANOVA tests were used in all cases, except for dopamine, for which Kruskal–Wallis tests were employed. Statistical significance was recognized when $p < 0.05$. Statistical significance is represented as follows: * $p < 0.05$.

Table S3 Determination of neurotransmitters in the frontal cortex

	Groups			*Post hoc* test		
Neurotransmitter (pg/mL)	Control n=12	14-PI n=6	21-PI n=6	14-PI vs. Control	21-PI vs. Control	14-PI vs. Control
D	15.9 ± 2.1	11.6 ± 2.7	13.3 ± 3.9	*	ns	ns
E	7.7 ± 1.7	5.5 ± 1.4	5.5 ± 1.8	ns	ns	ns
NE	14.9 ± 1.8	12.5 ± 2.8	12.1 ±2.4	ns	ns	ns
5-HT	25.3 ± 4.4	14.6 ± 2.5	23.4 ± 7.4	***	ns	*

In all cases the mean ± standard deviation is described. 14-PI= 14 days post infection, 21-PI= 21 days post infection. An ANOVA test was used in all cases, except for epinephrine, in which the Kruskal–Wallis test was applied. Statistical significance was recognized when $p < 0.05$. Statistical significance is represented as follows: * $p < 0.05$; *** $p < 0.001$.

Table S4. Determination of neurotransmitters in the hippocampus

	Groups			*Post hoc* test		
Neurotransmitter (pg/mL)	Control n=12	14-PI n=6	21-PI n=6	14-PI vs. Control	21-PI vs. Control	14-PI vs. Control
D	91.3 ±32.7	29.1 ±3.4	83.4 ± 15.5	***	ns	**
E	2.4 ±0.6	2.2 ±0.6	2.3 ± 0.5	ns	ns	ns
NE	10.4 ± 1.2	7.1 ± 0.4	10 ± 1.5	****	ns	**
5-HT	22.3 ±4.2	8.2 ±2.4	22.4 ± 4.7	**	ns	**

In all cases the mean ± standard deviation is described. 14-PI= 14 days post infection, 21-PI= 21 days post infection. ANOVA tests were used in all cases, except for serotonin, in which the Kruskal–Wallis tests were utilized. Statistical significance was recognized when $p < 0.05$. Statistical significance is represented as follows: ** $p < 0.01$; *** $p < 0.001$; **** $p < 0.0001$.

Table S5. Inflammatory profile in Balb/c mice infected with *B. abortus* 2308

	Groups			*post hoc* test		
Cytokine (pg/mL)	Control n=12	14-PI n=6	21-PI n=6	14-PI vs. Control	21-PI vs. Control	14-PI vs. Control
TNF-α	6.9 ± 3.2	18.3 ± 4.5	6.57 ± 1.8	****	ns	****
IL-6	7.5 ± 1.5	14.6 ± 3.3	4.6 ± 2.2	****	**	****
IL-12	15.3 ± 3.6	28.9 ± 8.6	21.9 ± 6.8	****	*	ns
IL-10	35.1 ± 13.5	16.4 ± 9.9	16.59 ± 11.4	***	***	ns
IFN-γ	15.3 ± 4.9	245.8 ± 88.7	16.0 ± 5.0	****	ns	****

In all cases the mean ± standard deviation is described. 14-PI= 14 days post infection, 21-PI= 21 days post infection. Kruskal–Wallis tests were used in all cases, except for IFN-γ, in which ANOVA tests were used. Statistical significance was recognized when $p < 0.05$. Statistical significance is represented as follow: ** $p < 0,01$; *** $p < 0,001$; **** $p < 0,0001$; ns = no significance.
